# Highly Aligned, Interconnected Porous Scaffolds via Photopolymerization of Acrylated Epoxidized Soybean Oil Containing Thermoreversible Terpenes as Porogens

**DOI:** 10.3390/ma19112206

**Published:** 2026-05-23

**Authors:** Jae-Uk Song, Jae-Hyung Park, Young-Hag Koh

**Affiliations:** 1 School of Biomedical Engineering, Korea University, Seoul 02841, Republic of Korea; jx0582@korea.ac.kr (J.-U.S.); jhbest210@korea.ac.kr (J.-H.P.); 2 Interdisciplinary Program in Precision Public Health, Korea University, Seoul 02841, Republic of Korea

**Keywords:** acrylated epoxidized soybean oil, terpene, phase separation, photopolymerization, porosity

## Abstract

Acrylated epoxidized soybean oil (AESO) is a bio-based, biocompatible, and biodegradable photopolymerizable resin that exhibits shape-memory behavior, making it attractive for a wide range of biomaterial applications. Despite various strategies to fabricate porous AESO scaffolds for tissue regeneration, achieving high pore interconnectivity remains challenging. Herein, we demonstrate the utility and versatility of thermoreversible terpenes as porogens in AESO to enable the formation of highly aligned and interconnected pore architectures. More specifically, a blend of 90 wt% camphene and 10 wt% camphor was employed as the terpene system, since it could be completely melted at 70 °C, uniformly mixed with liquid AESO, and subsequently crystallized at −20 °C. This process generated a bicontinuous network comprising terpene crystals and liquid AESO, thereby enabling efficient UV photopolymerization of AESO. Following terpene removal via freeze-drying, highly aligned pore networks with excellent pore interconnectivity were obtained, which are hardly achievable using conventional liquid or solid porogens. The porosity and mechanical properties of the AESO scaffolds were tuned by adjusting terpene content. Porosity increased from 61.5 to 81.5% as terpene content rose from 60 to 80 vol%. As a result, tensile strength decreased from 0.29 ± 0.045 to 0.17 ± 0.017 MPa, while elongation at break increased from 20.2 ± 4.9 to 35.5 ± 1.34%. Furthermore, this approach is compatible with vat photopolymerization (VP), a 3D printing technique. As a proof of concept, dual-scale porous AESO scaffolds, composed of unidirectional channels surrounded by highly aligned porous frameworks, were successfully fabricated. These results indicate that a variety of dual-scale porous AESO scaffolds, with greatly enhanced mechanical properties at given porosities coupled with outstanding tissue regeneration, can be produced through VP using terpene porogens, in contrast to conventional porous scaffolds comprising uniform porous frameworks.

## 1. Introduction

Acrylated epoxidized soybean oil (AESO) has emerged as a promising material in biomedical engineering, particularly for tissue regeneration and drug delivery systems [1,2]. As a derivative of soybean oil, AESO exhibits excellent cytocompatibility with surrounding tissues when implanted into the human body, along with biodegradability and high mechanical flexibility [2,3,4,5,6]. Furthermore, AESO can undergo rapid photopolymerization under UV irradiation, enabling precise control over material shaping [7,8]. This feature also makes it possible to fabricate patient-specific three-dimensional constructs with complex geometries using 3D printing techniques [8,9,10,11].

To facilitate tissue regeneration, it is necessary to incorporate 3D pore networks within AESO constructs [12,13,14,15]. These networks provide critical pathways for the transport of physiological fluids—such as blood, growth factors, oxygen, and nutrients—while simultaneously supporting the infiltration and formation of new tissue [15,16]. However, the introduction of pores significantly compromises the mechanical properties of porous AESO constructs, with the extent of reduction being strongly influenced by pore geometry [17,18,19]. In addition, the high viscosity of AESO resin limits the applicability of conventional techniques for manufacturing porous polymeric constructs [20,21]. A promising approach involves emulsifying hydrophilic water droplets within the hydrophobic AESO resin, followed by UV-induced photopolymerization of AESO and subsequent removal of the water droplets through drying or freeze-drying [15,22,23]. Alternatively, solid leachable particulates (e.g., salt or sugar) can be incorporated into AESO, followed by photopolymerization and subsequent particle leaching using water [24,25]. However, both methods employing pore-forming agents (porogens) often result in narrow interconnections between pores, while pore geometry remains uncontrollable, being dictated by the shapes of water droplets and leachable particles.

On the other hand, using terpenes as pore-forming agents enables the construction of three-dimensionally interconnected porous structures within polymers [26,27,28,29]. More specifically, terpenes such as camphene (C_10_H_16_) and camphor (C_10_H_16_O), which are solid at room temperature, can be melted above their melting points—approximately 44–48 °C for camphene [27,30] and ~175–177 °C for camphor [30,31]—and then crystallize as dendrites with tree-like morphologies upon cooling. The terpene crystals can be readily and completely removed via sublimation by freeze-drying due to their vapor pressure, resulting in highly interconnected pore networks with a high degree of pore alignment. In addition, terpenes are naturally derived from plants, environmentally friendly, and non-cytotoxic, making them a promising pore-forming agent for producing porous polymer constructs for biomedical applications [27,31,32]. Molten terpenes can also serve as a solvent for dissolving specific thermoplastics, such as polypropylene [31,33,34], ultra-high-molecular-weight polyethylene [35,36], polystyrene [27,37], and poly(ethyl methacrylate) [27], at elevated temperatures, followed by crystallization. Furthermore, molten terpenes can be uniformly mixed with liquid photopolymerizable monomers (photopolymers), such as tetraethylene glycol dimethacrylate (TEGDMA) [38] and polyethylene glycol diacrylate (PEGDA) [39]. These blends can undergo terpene crystallization during freezing at low temperatures, followed by photopolymerization of the monomers to form solid polymer networks.

**Figure 1:**
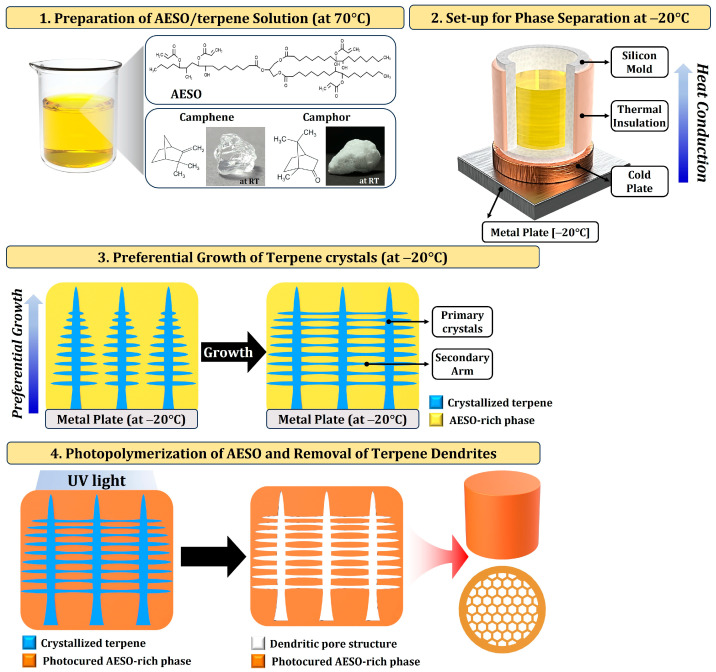
Schematic illustration of the fabrication of highly aligned, interconnected porous scaffolds via photopolymerization of AESO containing thermoreversible terpenes as porogens: preparation of a terpene/AESO solution at 70 °C, phase separation characterized by unidirectional growth of terpene crystal at −20 °C, and photopolymerization under UV light using VP process, and pore formation via camphene sublimation.

This study reports the fabrication of porous AESO scaffolds featuring highly aligned and interconnected pore networks, achieved by employing a terpene blend of 90 wt% camphene and 10 wt% camphor as a porogen. In this blend, camphene provides a moderate melting point, while camphor contributes sufficient mechanical strength. The schematic diagram of our approach is illustrated in Figure 1. Predetermined amounts of camphene, camphor, and AESO were added into vials and uniformly mixed at 70 °C. To tailor the porosity and mechanical properties of porous AESO scaffolds, various terpene contents (60, 70, and 80 vol%) were employed. The resulting terpene–AESO blends were cast into polymeric molds and then placed onto Cu plates maintained at approximately −20 °C for 1 min to induce phase separation, characterized by crystallization and preferential growth of the terpene, forming primary and secondary arms oriented parallel and normal to the direction of heat conduction, respectively. Immediately after freezing, the samples were exposed to UV light for 10 min to facilitate the photopolymerization of AESO. The resulting samples were freeze-dried to remove terpene crystals, yielding two types of highly aligned pore networks with interconnection between the pores. The porous structures and mechanical properties of the porous AESO scaffolds were systematically characterized. Furthermore, to demonstrate the versatility of our approach, dual-scale porous AESO scaffolds, composed of unidirectional channels surrounded by highly aligned porous frameworks, were fabricated using vat photopolymerization (VP) as a 3D printing technique.

## 2. Materials and Methods

### 2.1. Starting Materials

**Figure 2:**
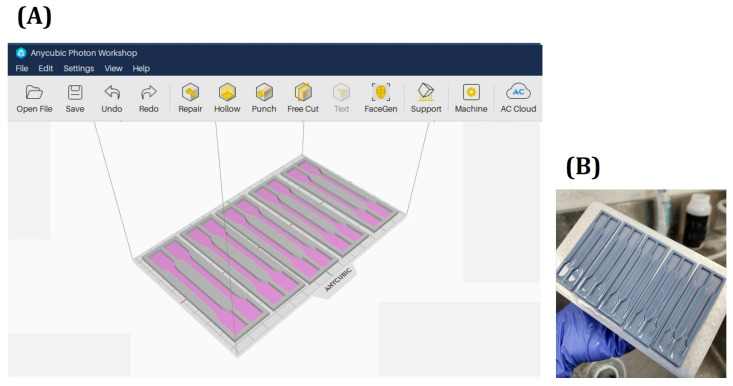
(**A**) Layout of dog-bone-shaped molds in the slicing software and (**B**) 3D-printed molds used for preparing specimens for tensile test. In Figure 2 (A), the purple and gray regions represent the recessed channel areas of the mold and the surrounding mold structure respectively.

Acrylated epoxidized soybean oil (AESO) was used as the base photocurable monomer (cf., Figure 1) and 2,4,6-trimethylbenzoyl diphenylphosphine oxide (TPO) served as the photoinitiator. Camphene (95% purity) and camphor (96% purity) were employed as terpene-based porogens (cf., Figure 1). All chemicals were purchased from Sigma-Aldrich (St. Louis, MO, USA) and used as received without further purification. The physicochemical properties of camphene and camphor, as provided in the supplier’s technical datasheets, are summarized in Table 1.

### 2.2. Preparation of AESO/Terpene Solutions

To ensure complete melting of terpenes at 70 °C, a blend of 90 wt% camphene and 10 wt% camphor was employed, considering its melting point [39]. Three types of AESO/terpene solutions with varying terpene contents (60, 70, and 80 vol%) were prepared by mixing molten terpene with liquid AESO at 70 °C in a dry oven, as summarized in Table 2. For the photopolymerization process, the photoinitiator TPO was added at 2 wt% relative to the AESO content. The optical appearance of AESO/terpene solutions at 70 °C was recorded with a digital camera to confirm the formation of homogeneous solutions.

### 2.3. Fabrication of Porous AESO Scaffolds via Phase Separation-Assisted Photopolymerization

A schematic illustration of the fabrication of highly aligned, interconnected porous scaffolds via photopolymerization of AESO containing thermoreversible terpenes as porogens is illustrated in Figure 1. Three types of AESO/terpene solutions with varying terpene contents (60, 70, and 80 vol%) prepared at 70 °C were cast into preheated plastic cylindrical molds to prevent excessively rapid phase separation. Immediately after casting, the mold openings were sealed with lids to minimize evaporation of the terpene phase during subsequent cooling. The solution-filled molds, with Cu plates at their bottoms, were placed onto a cold plate maintained at approximately −20 °C for about 1 min to induce phase separation of AESO/terpene solutions, characterized by crystallization and preferential growth of terpene crystals. Subsequently, the samples were transferred to a UV curing chamber with a power output of 16 watts at a peak wavelength of 385 nm and exposed to UV light for 3 min to fully photopolymerize the AESO phase. The resulting solid, rigid samples were removed from the molds and then freeze-dried for 24 h to completely remove the terpene crystal via sublimation.

### 2.4. Bulk Density and Overall Porosity of Porous AESO Scaffolds

The overall porosity of the porous AESO scaffolds was estimated from their bulk density. Bulk density was determined from the mass and geometric volume of the specimens after freeze-drying. The diameter and height of the cylindrical specimens were measured with a digital caliper. The specimen volume was then calculated from these dimensions, and the bulk density ($\rho_{b}$) was obtained by dividing the measured mass (*m*) by the calculated volume (*V*) as follows:

(1) $$\rho_{b}\;\left[g/{cm}^{3}\right]=m/V$$

Relative density ($\rho_{rel}$) and overall porosity (*P*) were calculated from the bulk density ($\rho_{b}$) and theoretical density ($\rho_{t}$) of AESO as follows:

(2) $$\rho_{rel}\;\left[\%\right]=100\cdot (\rho_{b}/\rho_{t})$$

(3) $$P\;\left[\%\right]=100-\rho_{rel}$$

### 2.5. Morphological and Microstructural Characterization of Porous AESO Scaffolds

The pore morphology and microstructure of the freeze-dried porous AESO scaffolds, prepared from AESO/terpene solutions with varying terpene contents (60, 70, and 80 vol%), were examined using field-emission scanning electron microscopy (FE-SEM, JSM-6701F, JEOL Ltd., Tokyo, Japan). Cylindrical specimens were carefully sectioned perpendicular to the phase separation direction to observe the formation of highly aligned, interconnected pores, which replicated the preferential growth of terpene crystals. For FE-SEM observations, specimen surfaces were sputter-coated with a thin Au layer at a current of 20 mA for 120 s using a sputter coater (Model 108 Auto, Cressington Scientific Instruments, Watford, UK). FE-SEM images were acquired at an accelerating voltage of 10.0 kV and at appropriate magnifications to resolve both the overall pore morphology and the microstructure of the AESO frameworks.

### 2.6. Mechanical Behavior and Property Evaluation of Porous AESO Scaffolds

The mechanical properties of porous AESO scaffolds, prepared from AESO/terpene solutions with varying terpene contents (60, 70, and 80 vol%), were examined using tensile and compression testing. For the tensile strength tests, polymeric molds were fabricated using a commercial VP printer (Photon Mono 2, Anycubic, Shenzhen, China) for preparing dog-bone-shaped specimens (Figure 2A,B). The geometry and dimensions of specimens, designed in accordance with ISO 527-2 (type 5A) [40], were precisely modeled using a 3D design program (Autodesk Inventor Professional 2025, Autodesk Inc., San Rafael, CA, USA). Rigid molds were then fabricated using a commercially available photosensitive resin (Standard Resin, Anycubic), where a layer thickness of 50 µm and an exposure time of 2 s were employed under a light intensity up to 4200 μW/cm^2^. After printing, the molds were rinsed with isopropanol (IPA) to remove uncured resin and post-cured in a UV curing chamber. They exhibited high dimensional accuracy (a thickness of ~1.8 mm, a gauge width of ~3.7 mm, and a gauge length of ~65 mm) and sufficient strength, ensuring that the porous AESO specimens could be removed without damage. Three types of AESO/terpene solutions were cast into the molds and then placed at −20 °C for about 1 min to induce phase separation, followed by photopolymerization in the UV curing chamber. The photopolymerized samples were subsequently removed from the molds and freeze-dried to obtain porous AESO specimens. In a similar manner, cylindrical samples with a diameter of 12 mm and a height of 6 mm were prepared using plastic molds for compressive strength tests.

Tensile tests (ISO 527-2) [40] and compression tests (ASTM D695-15) [41] were conducted using a universal testing machine (ST-1000, Salt Co., Ltd., Incheon, Republic of Korea) at a constant crosshead speed of 0.2 mm/min. During the tests, stress was recorded as a function of strain to calculate tensile strength, elongation at break, and compressive modulus. For the tensile tests, dog-bone-shaped specimens were unidirectionally elongated until fracture occurred. For compression tests, cylindrical samples were compressed unidirectionally, with the load applied parallel to the direction of the elongated pores. For each AESO/terpene composition (60, 70, and 80 vol% terpene), at least three specimens were tested to determine average values and standard deviations.

### 2.7. Fabrication of Dual-Scale Porosity AESO Scaffolds Using VP

**Figure 3:**
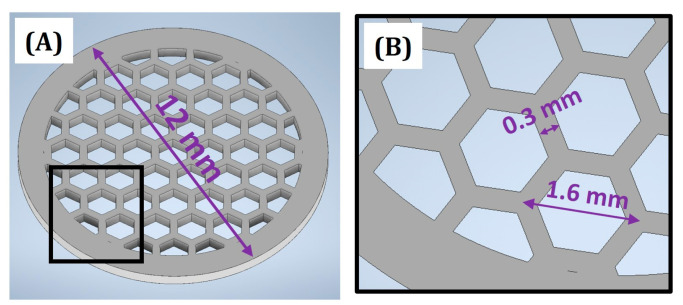
CAD model of dual-scale porosity composed of unidirectional channels surrounded by porous frameworks: (**A**) overall structure with unidirectional channels and (**B**) geometry and dimensions of the channels and porous AESO frameworks.

To demonstrate the utility AESO/terpene solutions for VP, dual-scale porosity composed of unidirectional channels surrounded by aligned porous frameworks was fabricated. The CAD models illustrating the overall structure with unidirectional channels, as well as the geometry and dimensions of the channels and porous AESO frameworks, are shown in Figure 3A,B. A scaffold with a diameter of 12 mm, a cell pitch of 1.6 mm, and a strut thickness of 0.3 mm was modeled using a 3D CAD program (Autodesk Inventor Professional 2025, Autodesk Inc., San Rafael, CA, USA). A single, uniform layer of the AESO/terpene solution with a terpene content of 60 vol% was selectively photopolymerized for 24 s using a digital light processing (DLP) module (PRO4710, Wintech Digital Systems Technology Corp., San Jose, CA, USA), which illuminated UV light at a peak wavelength of 405 nm. Subsequently, uncured regions were rinsed, and the specimens were freeze-dried to remove the terpene crystals. The resulting scaffolds were examined by optical microscopy (OM) and FE-SEM.

## 3. Results and Discussions

### 3.1. Mixing Behavior of AESO/Terepne Solutions at 70 °C

**Figure 4:**
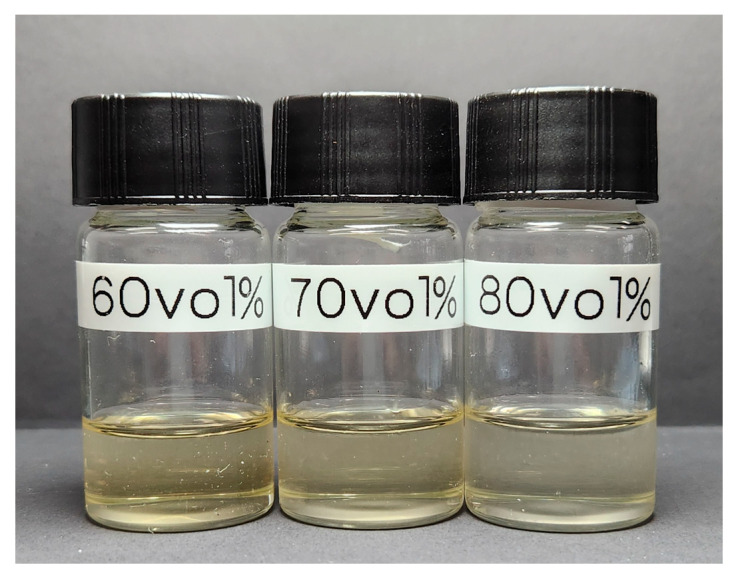
Optical images of AESO/terpene solutions at 70 °C with varying terpene contents (60, 70, and 80 vol%).

To fully exploit AESO/terpene solutions to create a highly aligned and interconnected porous structure, terpenes must be completely melted and homogeneously mixed with liquid AESO monomer at moderate temperatures (e.g., 70 °C, as used in this study) to enable favorable casting. In addition, AESO/terpene solutions should undergo phase separation at processable low temperatures (e.g., −20 °C, as used here), where molten terpene can crystallize and then preferentially grow along the direction of heat conduction. For this purpose, a blend of 90 wt% camphane and 10 wt% camphor was employed as a terpene-based porogen. More specifically, the addition of relatively hard camphor to soft camphene can enhance rigidity and strength after phase separation. However, the melting point of the camphene–camphor system increases linearly with increasing camphor content [39], thereby limiting the maximum camphor fraction applicable in this approach. Regardless of terpene content (60, 70, and 80 vol%), all AESO/terpene solutions exhibited clear states without any local turbidity or segregation at 70 °C (Figure 4). This observation indicates that molten terpene can be homogeneously mixed with liquid AESO monomer. Although some portions of solid camphene and camphor can be dissolved in liquid AESO at 70 °C, this behavior would have negligible influence on the phase separation behavior of AESO/terpene solutions at the very low temperature of −20 °C.

### 3.2. Optical Appearance of As-Photopolymerized and Freeze-Dried Samples

**Figure 5:**
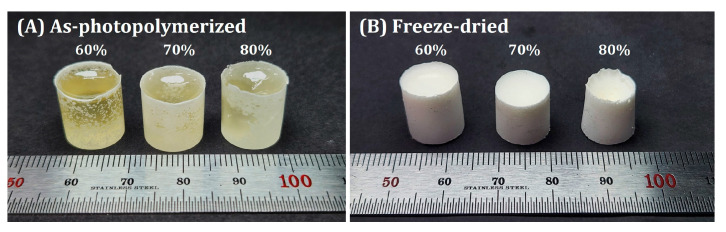
Optical images of (**A**) as-photopolymerized samples and (**B**) freeze-dried samples produced with varying terpene contents (60, 70, and 80 vol%).

After photopolymerization, no noticeable change in optical appearance was observed across all compositions with varying terpene contents (60, 70, and 80 vol%), as shown in Figure 5A. High transmittance was obtained since the samples consisted of photopolymerized AESO and terpene crystals, while the yellowish color was simply attributed to the photopolymerized AESO. However, semi-transparent outer surfaces were observed, presumably due to the skin layer of terpene crystals that rapidly crystallized at the mold wall during the early stage of phase separation at −20 °C. However, following freeze-drying, all samples turned opaque, as shown in Figure 5B. This change indicates that terpene crystals can be removed via sublimation, thereby creating pores within the AESO matrix and causing significant refractive index mismatch. It should be noted that no noticeable distortion or cracks were observed, confirming the utility of terpene as an effective porogen.

### 3.3. Porous Structure of Porous AESO Scaffolds and Microstructure of Frameworks

**Figure 6:**
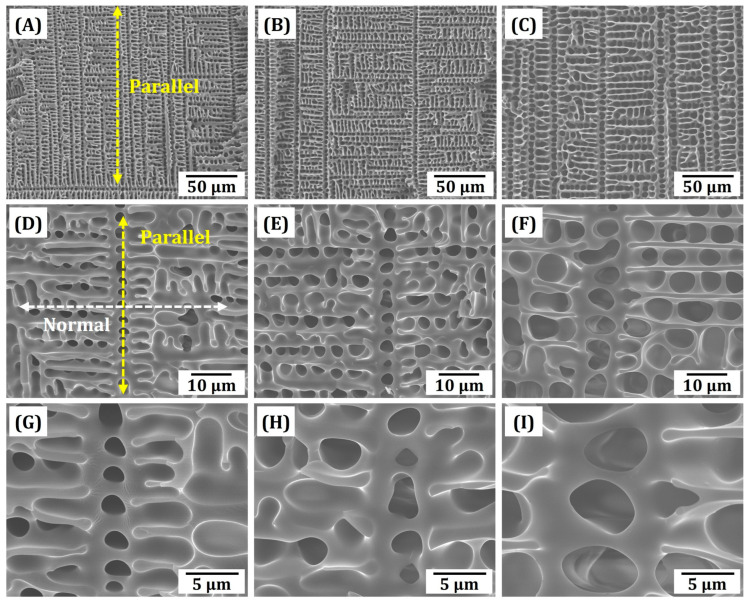
FE-SEM images of porous AESO scaffolds produced with varying terpene contents: 60 vol% (**A**,**D**,**G**), 70 vol% (**B**,**E**,**H**) and 80 vol% (**C**,**F**,**I**). “Parallel” and “normal” denote the directions parallel and normal to the dendritic growth of terpene crystals.

The porous structure of AESO scaffolds and the microstructure of their frameworks were characterized by FE-SEM (Figure 6A–I). Regardless of terpene content (60, 70, and 80 vol%), all scaffolds exhibited highly uniform porous structures throughout (Figure 6A–C). Moreover, highly aligned pores were formed due to the preferential growth of terpene crystals along the direction of heat conduction from the bottom to the top during phase separation at −20 °C. Specifically, terpene crystals can grow dendritically, producing unidirectionally aligned primary arms, while secondary arms simultaneously branch out from the primary arms in the normal direction. This dendritic growth resulted in unique porous structures (Figure 6D–F), where two types of highly aligned pore networks were aligned both parallel and normal to the dendritic growth of terpene crystals. These pore networks were interconnected. It should be noted that highly aligned porous structures can offer significantly greater compressive strength than uniform porous structures when loaded parallel to the pore direction, and they can effectively guide direction-dependent regeneration of specific tissues such as nerves. All scaffolds exhibited dense and smooth frameworks (Figure 6G–I). This observation indicates that the terpene phase can undergo phase separation from the AESO-terpene solution and grow dendritically without altering the AESO phase. On the other hand, both pore size and framework thickness increased with higher terpene content, as is commonly observed in thermally induced phase separation of polymer solutions [38,39]. It should be noted that all scaffolds exhibited sufficiently large, aligned pore networks, which are expected to effectively guide the migration of cells in processes such as nerve regeneration, thereby leading to biomimetic tissue regeneration.

### 3.4. Overall Porosity of Porous AESO Scaffolds

**Figure 7:**
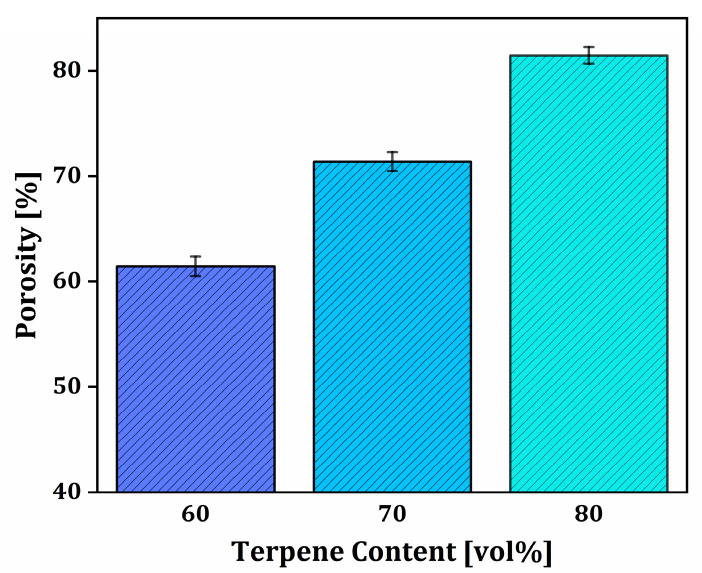
Overall porosity of AESO scaffolds fabricated with different terpene contents (60, 70, and 80 vol%).

The overall porosity of AESO scaffolds fabricated with varying terpene contents (60, 70, and 80 vol%) was estimated by comparing their measured density and the theoretical density of photopolymerized AESO reported in the literature. The results are presented in Figure 7. The measured porosities were 61%, 71%, and 81 vol% for terpene contents of 60 vol%, 70%, and 80%, respectively. This finding suggests that molten terpene can almost completely undergo phase separation from AESO/terpene solutions at a very low temperature of −20 °C, although partial dissolution occurs in AESO at 70 °C. It should be noted that the porosity of porous AESO scaffolds can be tailored for specific target tissues simply by adjusting the terpene content. However, increasing terpene content compromises the mechanical integrity of porous AESO scaffolds, thereby limiting their suitability for clinical applications.

### 3.5. Mechanical Behaviors and Properties of Porous AESO Scaffolds

**Figure 8:**
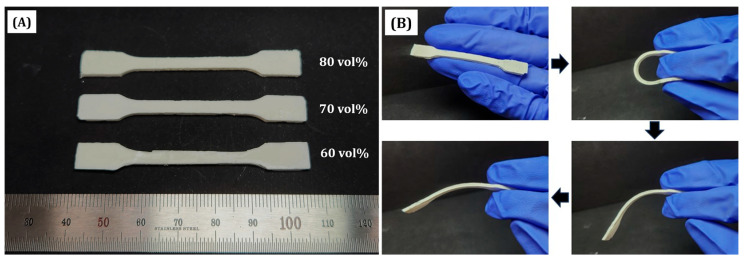
Optical images illustrating (**A**) three types of specimens fabricated with different terpene contents (60, 70, and 80 vol%) and (**B**) the flexibility of a specimen undergoing bending followed by recovery.

To examine the potential of highly aligned porous AESO scaffolds for tissue regeneration, their mechanical behaviors and properties were examined by tensile and compression testing. For tensile strength measurements, three types of specimens, conforming to the ISO 527-2 type 5A geometry [40], were fabricated with varying terpene contents (60, 70, and 80 vol%). All specimens exhibited well-defined geometry and dimensions (Figure 8A), owing to the favorable casting of AESO/terpenes solutions, which possessed sufficiently low viscosity and high flowability at 70 °C when introduced into 3D printed polymeric molds. All specimens exhibited high flexibility, and a representative OM image of a specimen with a terpene content of 60 vol% is illustrated in Figure 8B. The specimen could be bent to large curvatures without visible cracking, and it largely recovered its original shape after unloading. With increasing terpene content, the specimens became noticeably more compliant, reflecting the higher overall porosity. This finding suggests that the highly aligned porous AESO scaffolds produced in this study can serve as deformable scaffolds capable of conforming to the 3D geometry of target tissues.

**Figure 9:**
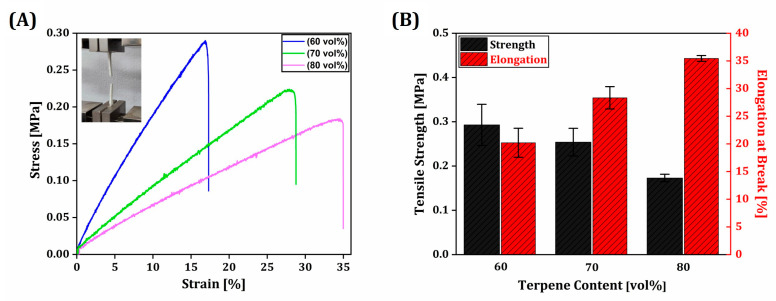
(**A**) Representative tensile stress–strain curves of porous AESO scaffolds with varying terpene contents (60, 70, and 80 vol%) and (**B**) tensile strength and elongation at break as a function of terpene content (*n* = 4). The inset in Figure 9A shows the fractured specimen with a terpene content of 60 vol%.

The mechanical properties of porous AESO scaffolds with varying terpene contents (60, 70, and 80 vol%) were evaluated by tensile testing. Their representative stress–strain curves are present in Figure 9A. All samples exhibited an almost linear regime at small strains, characteristic of elastic deformation, followed by gradual non-linear deformation up to the maximum stress, after which the load dropped upon fracture. All specimens fractured near their center and a representative OM image of a fractured specimen with a terpene content of 60 vol% is shown in the inset of Figure 9A. As the terpene content increased from 60 to 80 vol%, tensile strength decreased from 0.29 ± 0.045 to 0.17 ± 0.017 MPa, while elongation at break increased from 20.2 ± 4.9 to 35.5 ± 1.34% (Figure 9B), attributed to the increase in porosity (cf., Figure 7). It should be noted that highly aligned pore networks produced in this study can more effectively withstand applied tensile loads, thereby offering superior mechanical properties to uniform porous structures.

**Figure 10:**
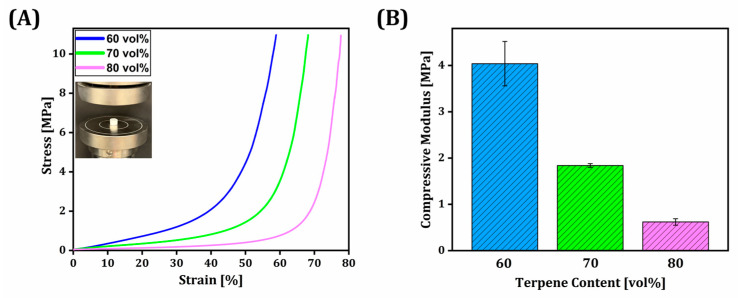
(**A**) Representative compressive stress–strain curves of porous AESO scaffolds with varying terpene contents (60, 70, and 80 vol%) and (**B**) compressive modulus as a function of terpene content (*n* = 4). The inset in Figure 10A shows the specimen with a terpene content of 60 vol% positioned between two platens for compression test.

Compression testing was conducted to evaluate the resistance of porous AESO scaffolds to compressive loads, which are typically encountered when used as scaffolds for tissue regeneration. To this end, specimens were positioned between two platens and then unidirectionally compressed along the axis parallel to the elongated pores (inset in Figure 10A. Regardless of terpene content (60, 70, and 80 vol%), all specimens exhibited the typical compressive stress–strain response of highly porous ductile polymers (Figure 10A). Compressive stress increased almost linearly with strain, followed by a steep rise due to the collapse of pores and densification of AESO walls. However, as the terpene content increased from 60 to 80 vol%, the compressive modulus decreased from 4.04 ± 0.47 to 0.62 ± 0.07 MPa (Figure 10B), which is attributed to the increase in porosity.

Table 3 summarizes the measured porosity and mechanical properties of porous AESO scaffolds with varying terpene contents (60, 70, and 80 vol%). As the terpene content increased, porosity increased, since pores were formed as replicas of terpene crystals generated through phase separation of AESO/terpene solutions. Consequently, tensile strength and compressive modulus decreased, whereas elongation at break increased. Notably, these values remain comparable to those of soft tissues due to the highly elongated porous structures, even at high porosity. Furthermore, the mechanical properties of porous AESO scaffolds can be tailored for specific target tissues simply by adjusting the terpene content.

### 3.6. VP of Dual-Scale Porosity AESO Scaffolds

**Figure 11:**
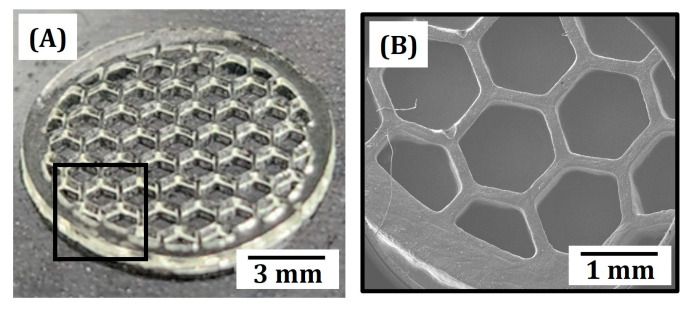
(**A**) Optical image and (**B**) FE-SEM image of a dual-scale porous AESO scaffold fabricated by VP using a terpene content of 60 vol%.

To demonstrate the utility of AESO/terpene solutions for VP, dual-scale porous AESO scaffolds composed of unidirectional channels separated by highly aligned porous frameworks were fabricated using a terpene content of 60 vol%. The scaffolds exhibited well-defined unidirectional channels arranged in a hexagonal pattern (Figure 11A). No noticeable distortion was observed after either photopolymerization or freeze-drying. Furthermore, uniform and thin AESO frameworks were created throughout the scaffold (Figure 11B) despite their porous structure. These findings suggest that AESO/terpene solutions are suitable for VP for the fabrication of dual-scale porous scaffolds, which can provide versatile designs with tunable mechanical properties for target applications, as well as excellent tissue generation ability owing to their 3D channels and interconnected pore networks within the frameworks. It should be noted that the elongated porous structure is expected to effectively guide the migration of cells, thereby facilitating tissue regeneration in processes such as nerve regeneration, for example. However, this potential should be systematically verified through in vitro and in vivo studies to optimize both macroporous and microporous structures of dual-scale porous scaffolds. The use of thermoreversible terpene as a porogen is applicable to a variety of photopolymerizable monomers, thereby offering opportunities to fabricate various dual-scale porous materials with different mechanical properties and biodegradation rates.

## 4. Conclusions

Porous AESO scaffolds with highly aligned and interconnected pore architectures were fabricated using terpene as a thermoreversible porogen in photopolymerizable AESO monomer. Terpene, molten and homogeneously mixed with AESO at 70 °C, crystallized and grew dendritically at −20 °C, forming primary and secondary arms oriented parallel and normal to the direction of heat conduction, respectively. Consequently, two types of highly aligned pore networks were generated after the removal of terpene crystals, which had been embedded within the photopolymerized AESO frameworks. As the terpene content increased from 60 to 80 vol%, porosity increased, while tensile strength decreased and elongation at break improved. However, all scaffolds exhibited reasonable tensile strengths (0.29–0.17 MPa) and elongation at break values (20.2–35.5%) at high porosities (61.5–81.5 vol%), indicating their suitability for use as tissue regeneration scaffolds. Furthermore, dual-scale porous scaffolds composed of unidirectional hexagonal channels surrounded by highly aligned porous frameworks were successfully fabricated by VP. These findings indicate that thermoreversible terpene can be effectively employed as a porogen to generate highly elongated, interconnected pore networks in AESO scaffolds, thereby offering significantly enhanced mechanical properties at a given porosity. In addition, the elongated porous structure is expected to guide and stimulate tissue regeneration when applied as a scaffold. However, further in vitro and in vivo investigations are required to optimize both macroporous and microporous structures of porous AESO scaffolds for enhanced tissue regeneration performance.

## Figures and Tables

**Table 1:** Physicochemical properties of camphene and camphor.

	Camphene	Camphor
Molecular formula	C_10_H_16_	C_10_H_16_O
Molar mass (g·mol^−1^)	≈136.24	≈152.23
Melting point (°C)	48–52	175–177
Density (g·mL^−1^)	0.85 (at 25 °C)	0.99 (at 25 °C)

**Table 2:** Compositions of AESO/terpene solutions with various terpene contents (60, 70, and 80 vol%), incorporating TPO as the photoinitiator.

Terpene Content [vol%]	AESO [wt%]	Camphene [wt%]	Camphor [wt%]	TPO [wt%]
60	43.7	49.9	5.5	0.9
70	33.4	59.3	6.6	0.7
80	22.7	69.1	7.7	0.5

**Table 3:** Measured porosity and mechanical properties of porous AESO scaffolds with varying terpene contents (60, 70, and 80 vol%).

Terpene Content [vol%]	Porosity [%]	Tensile Strength [MPa]	Elongation at Break [%]	Compressive Modulus [MPa]
60	61.5 ± 0.76	0.29 ± 0.045	20.2 ± 4.9	4.04 ± 0.47
70	71.0 ± 0.68	0.25 ± 0.03	28.3 ± 2.1	1.84 ± 0.04
80	81.5 ± 0.74	0.17 ± 0.017	35.5 ± 1.3	0.62 ± 0.07

## Data Availability

The original contributions presented in this study are included in the article. Further inquiries can be directed to the corresponding author.
